# Synergistic Thresholds Governing Performance Evolution in Red Mud-Fly Ash-Coal Gangue Ternary Solid Waste Concrete (RFCTSWC)

**DOI:** 10.3390/ma18163754

**Published:** 2025-08-11

**Authors:** Jin Qu, Yujie Tian, Jiale Liu, Runfang Zhou, Haitao Mao

**Affiliations:** 1 College of Agricultural Engineering, Shanxi Agricultural University, Jinzhong 030800, China; qujin@sxau.edu.cn (J.Q.); m13934054210@163.com (Y.T.); liujiale910@163.com (J.L.); zhourunfang@sxau.edu.cn (R.Z.); 2 College of Urban and Rural Construction, Shanxi Agricultural University, Jinzhong 030800, China; 3 Shanxi Smart Water-Saving Technology Innovation Center, Taiyuan 030000, China

**Keywords:** red mud, coal gangue aggregate, solid waste concrete, synergistic threshold, frost resistance durability

## Abstract

To address the environmental risks associated with large-scale stockpiling of red mud (RM) and coal gangue (CG) and the demand for their high-value utilization, this study proposes a ternary concrete system incorporating RM, fly ash (FA), and CG aggregate. The effects of RM content, FA content, CG aggregate replacement rate, and water-to-binder ratio on workability, mechanical properties, and frost resistance durability were systematically investigated through orthogonal experiments, with the underlying micro-mechanisms revealed by scanning electron microscopy (SEM) and energy dispersive spectroscopy (EDS). The results indicate that workability is predominantly governed by the water-to-binder ratio, while the micro-aggregate effect of FA significantly enhances fluidity. Mechanical properties are most significantly influenced by RM content; under a 20% CG aggregate replacement rate and a 0.45 water-to-binder ratio, an optimal compressive strength was achieved with a low content combination of RM and FA. Frost resistance deteriorated markedly with increasing RM and FA content, with the high-content group approaching the failure threshold after only 25 freeze–thaw cycles, occurring 50 and 125 cycles earlier than the medium- and low-content groups, respectively. Macro-micro results indicate a synergistic threshold at 20% red mud and 45% fly ash, yielding a compressive strength of 24.96 MPa. This value exceeds the 24.87 MPa of the 10% red mud + 45% fly ash group and the 21.90 MPa of the 10% red mud + 55% fly ash group. Microstructurally, this group also exhibits superior C-S-H gel uniformity and narrower crack widths compared to the others. Excessive incorporation of red mud and fly ash leads to agglomeration of unhydrated particles and increased porosity, aligning with the observed macroscopic strength degradation. This research identifies and quantifies the synergistic threshold governing RFCTSWC performance evolution, providing theoretical support for engineering applications of solid waste concrete.

## 1. Introduction

The total stockpile of industrial solid waste in China exceeds 60 billion tons [1], with coal gangue accumulations surpassing 5 billion tons [2]. RM stockpiles continue to grow at an annual production rate of approximately 100 million tons [3]. Such massive reserves face challenges, including low utilization rates and difficult disposal. The large-scale surface disposal of solid wastes such as red mud and coal gangue not only occupies substantial land resources [4] but also triggers geohazards, including landslides, soil erosion, and spontaneous combustion of waste piles [5]. These activities pose severe threats to surrounding ecosystems, including air, soil, and groundwater contamination [6]. In recent years, national policies have actively promoted the large-scale and high-value utilization of industrial byproducts [7]. Concrete, consumed globally at over 30 billion tons annually, emerges as a critical carrier for high-value red mud and coal gangue applications due to its inherent capacity for bulk solid waste incorporation [8]. Utilizing solid wastes like red mud, coal gangue, and fly ash as component materials in concrete production both reduces costs and consumes substantial amounts of solid waste. This research direction has become one of the active focal points in the current building materials industry.

Rich in reactive components such as SiO_2_, Al_2_O_3_, and CaO, RM exhibits pozzolanic properties that enable partial cement replacement in binder systems [9]. Studies confirm that concrete incorporating ≤20% RM maintains adequate workability and satisfies 28-day compressive strength requirements for engineering applications [10], while RM’s filling capacity effectively suppresses microcrack propagation within the matrix [11]. However, RM dosages exceeding 30% typically trigger workability deterioration and strength regression [12,13]. To mitigate these limitations, researchers propose performance enhancement strategies, including fiber reinforcement [14] or co-incorporation with fly ash [15]. Notably, the RM-FA synergy promotes enhanced hydration product formation and optimizes pore structure [16], while RM’s inherent alkalinity can effectively activate slag reactivity [17], revealing further pathways for synergistic solid waste utilization [18].

As one of the largest-volume industrial solid wastes in China, coal gangue possesses mineral components similar to natural aggregates, granting it broad prospects for utilization in building materials. However, coal gangue aggregate (CGA) is characterized by relatively high porosity, water absorption, and a significant content of elongated/flaky particles. These characteristics lead to a significant reduction in the mechanical properties and frost resistance durability of concrete when incorporated [19,20,21]. To address this issue, most current research focuses on aggregate modification techniques such as calcination [22], acid treatment [23], and fiber reinforcement [24,25]. Nevertheless, these modification processes are often complex, costly, and consequently face significant barriers to practical, large-scale application. Despite these challenges, utilizing unmodified or minimally modified CGA remains a crucial strategy for conserving natural resources and achieving large-scale recycling.

Fly ash, as an essential mineral admixture, possesses excellent pozzolanic reactivity and a micro-aggregate effect, making it an indispensable component in concrete. Recent studies have found that a binary synergistic system composed of 15% red mud and 15% coal gangue powder exhibits compressive strength comparable to cement concrete. The synergy between these two materials enhances compressive strength by filling concrete pores and generating new C-S-H gel structures [26]. Research by Koshy et al. [27] revealed that the improvement in macroscopic properties achieved by the binary powder system of red mud and coal gangue is significantly less effective than that of the ternary powder system comprising red mud, fly ash, and coal gangue. This multi-solid waste synergistic effect overcomes the limitations inherent to the performance influence of a single solid waste, providing a promising approach for the large-scale recycling of industrial solid waste.

Although some progress has been made in the individual or combined utilization of RM, FA, and CG in concrete, existing achievements notably lack systematic quantitative research on ternary synergistic effects. This is particularly evident regarding the workability, mechanical properties, and frost resistance durability of concrete under the synergistic action of the ternary system composed of RM, FA, and unmodified CGA. Furthermore, the reinforcement mechanism of the RM-FA synergy on the interfacial transition zone of unmodified CGA remains unclear and requires further clarification. Most critically, the synergistic threshold for the co-incorporation of RM and FA, as well as its relationship with the macro- and micro-properties of ternary solid waste concrete, urgently needs to be elucidated. The lack of a quantified relationship between the threshold and performance impedes the engineering application of ternary solid waste concrete.

Therefore, this study developed a ternary solid waste concrete system utilizing RM, FA, and CGA. Orthogonal tests quantified the influence weights of RM, FA, CGA, and water-binder ratio (W/B) on workability, compressive/splitting tensile strength, and frost resistance. Combined with SEM and EDS microstructural characterization of internal pore structures and hydration products, the mechanism of RM-FA co-incorporation in CGA concrete was investigated. This approach established the relationship between macro- and micro-properties and clarified the synergistic optimization threshold for solid wastes, thus providing a theoretical basis for the engineering design and performance regulation of RFCTSWC.

## 2. Materials and Methods

### 2.1. Raw Materials

#### 2.1.1. Cementitious Materials

**Figure 1:**
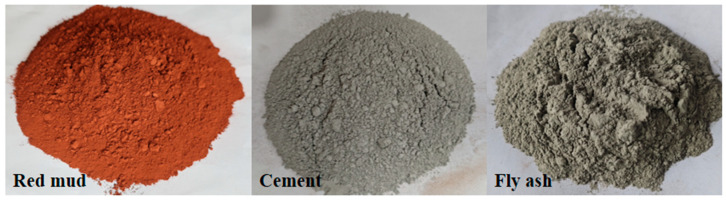
Red mud, cement, and fly ash used in the test.

The red mud used in this experiment was sourced from an alumina plant in Lüliang, China, with a density of 3.45 g/cm^3^ and a specific surface area of 776.4 m^2^/kg. The fly ash was Grade I fly ash obtained from Yangquan Mineral Powder Plant in Shanxi, exhibiting a density of 2.20 g/cm^3^ and a specific surface area of 450 m^2^/kg. The cement was P·O·42.5 ordinary Portland cement produced by Jinzhong Jianzhong Cement Products Co., Ltd., Jinzhong, China, featuring a density of 3.15 g/cm^3^ and a specific surface area of 360 m^2^/kg. The raw materials are shown in Figure 1, and their chemical compositions are listed in Table 1.

#### 2.1.2. Coarse and Fine Aggregates

**Figure 2:**
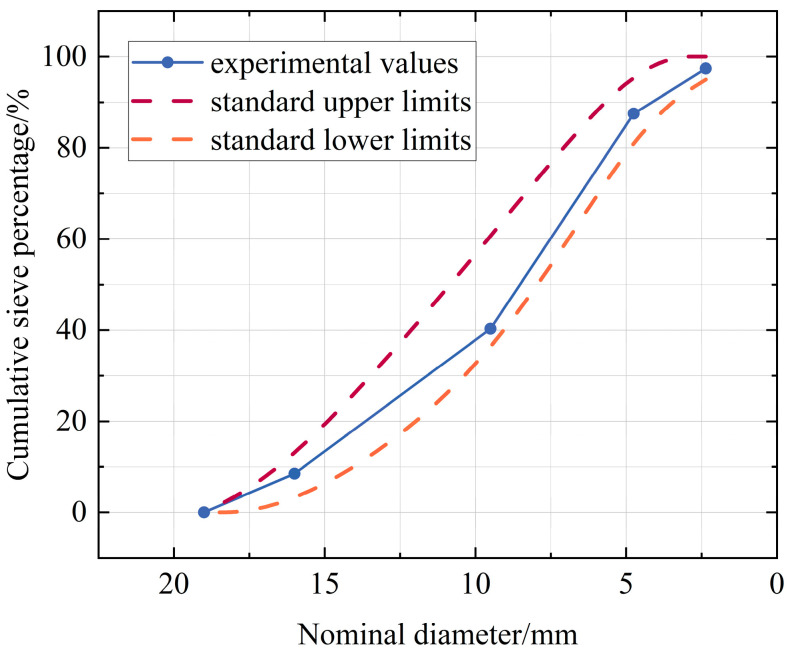
Cumulative sieve percentage of coal gangue aggregates (%).

The coarse aggregate consisted of crushed coal gangue and conventional crushed stone. CGA was crushed using a jaw crusher to achieve a particle size range of 5–16 mm. Secondary screening with slotted sieves was employed to control the content of needle-like/flaky particles below 10%. The cumulative sieve residue percentages are presented in Figure 2. Natural sand from the Jinzhong region was used as the fine aggregate, and its technical specifications are detailed in Table 2.

#### 2.1.3. Admixtures and Water

A polycarboxylate-based high-performance water-reducing agent produced by Henan Qimeng Chemical Co., Ltd. in Zhengzhou, China, has a water reduction rate of 25%. Tap water from the Jinzhong region of Shanxi Province was used as the mixing water for the concrete.

#### 2.1.4. Microscopic Characterization of Raw Materials

**Figure 3:**
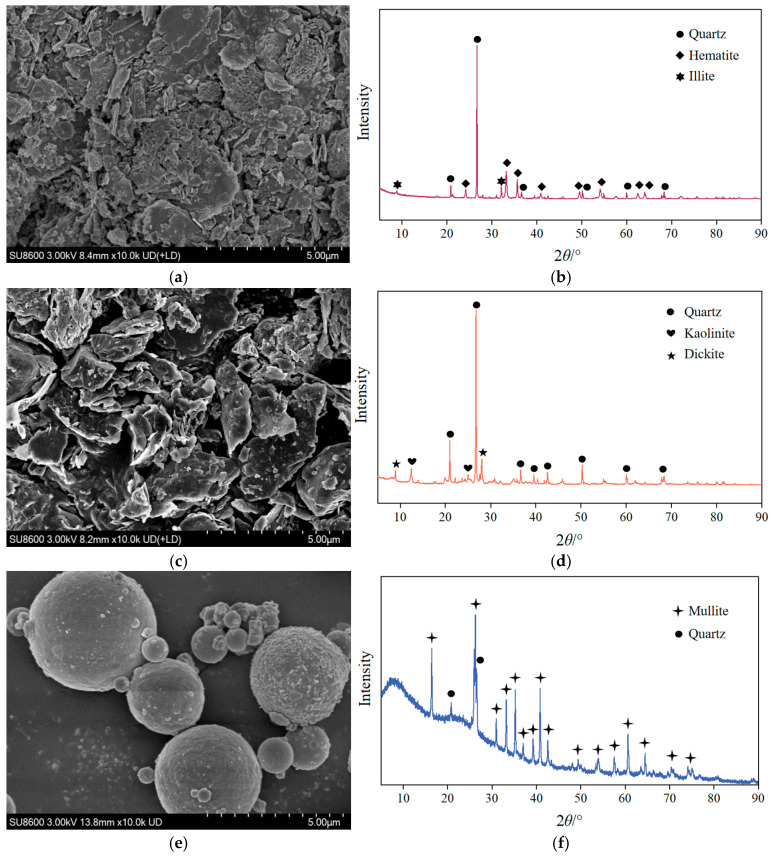
Microscopic characterization of raw materials. (**a**) SEM image of red mud. (**b**) XRD pattern of red mud. (**c**) SEM image of coal gangue. (**d**) XRD pattern of coal gangue. (**e**) SEM image of fly ash. (**f**) XRD pattern of fly ash.

The microscopic morphology and mineral composition of the RM, CG, and FA were characterized using SEM and X-ray diffractometry (XRD), as shown in Figure 3. At a magnification of 10,000×, the RM exhibited a plate-like and needle/rod-shaped structure, with quartz, hematite, and illite identified as its primary mineral phases. The CG displayed an irregular blocky structure with a rough surface, and its main mineral constituents were quartz, kaolinite, and dickite. The FA displayed a regular shape with a spherical structure, and its main mineral constituents were mullite and quartz.

### 2.2. Mix Proportions Design

Through existing research [12] and single-factor tests, it was found that when the RM content exceeds 30%, concrete experiences significant strength loss. Therefore, RM content was limited to below 30%. The performance differences of coarse aggregates directly affect concrete specimen properties. Insufficient CGA content fails to effectively improve solid waste utilization rates, while excessive amounts cause rapid deterioration of mechanical properties [28]. Consequently, the coal gangue replacement ratio was set at 20–60% of coarse aggregate content. Considering the critical influence of W/B on concrete performance, it was designated as a key factor. RM’s inherent viscosity and coal gangue’s high water absorption both reduce concrete workability, whereas FA’s microsphere effect effectively enhances workability [29] while reducing hydration heat and improving density. Thus, fly ash content was established as an influencing factor. Following China’s GB/T 1596-2017 [30] recommendation for high-volume incorporation of Grade I fly ash, the content was set at 35%, 45%, and 55%. Ultimately, a four-factor three-level orthogonal test L9(3^4^) was designed, with each factor corresponding to three variable values. The mix proportion design followed the requirements of JGJ55-2011 [31]. The specific factor level values and material quantities are shown in Table 3.

### 2.3. Sample Preparation

**Figure 4:**
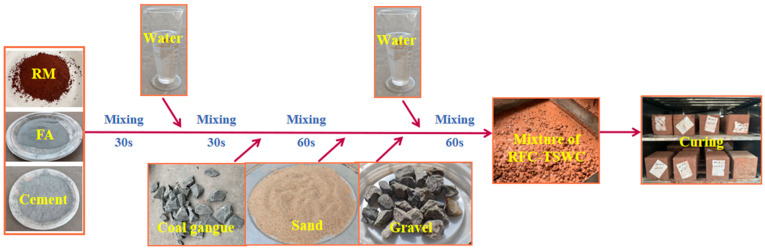
Flowchart for the preparation of concrete specimens.

Due to the higher water absorption of CGA, the conventional mixing sequence was modified. The dry powders of RM, cement, and FA were first mixed for 30 s. A portion of the mixing water was then added, followed by mixing for another 30 s. Subsequently, the CGA was introduced and mixed for 60 s to ensure a full and uniform coating of the binder materials on the aggregate surfaces. Sand, crushed stone, and the remaining water were then added, with mixing continued for a further 60 s. The fresh concrete was cast into 100 mm × 100 mm × 100 mm molds placed on a vibrating table and vibrated for 30 s; the surfaces were smoothed with a trowel. After demolding at room temperature for 48 h, the specimens were cured in a standard curing chamber maintained at 20 ± 2 °C and 95% relative humidity. The preparation flowchart for RFCTSWC specimens is presented in Figure 4.

### 2.4. Test Methods

#### 2.4.1. Macro Testing

**Figure 5:**
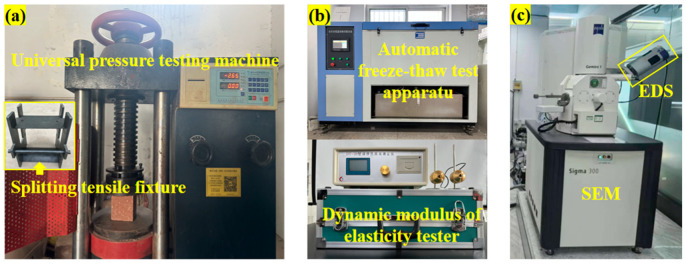
The instruments and equipment required for the test. (**a**) Mechanical property test. (**b**) Freeze–thaw test instrument. (**c**) Microscopic test instrument.

The slump of fresh concrete was measured according to the Chinese standard GB/T 50080-2016 [32] using a slump cone, steel ruler, and tamping rod. The compressive strength and splitting tensile strength of 100 mm × 100 mm × 100 mm cubic specimens, after a 28-day curing period, were tested in compliance with GB/T 50081-2019 [33]. The TSY-3000 automatic pressure testing machine manufactured by Zhongluchang Testing Machine Manufacturing Co., Ltd., Jinan, China was used for determination, as shown in Figure 5a. The splitting tensile strength test additionally required steel arc-shaped bearing blocks with a radius of 75 mm, plywood, and steel supports. The arithmetic mean of test results from three specimens in each group was taken as the representative strength for that group.

The freeze–thaw cycle test was conducted using the slow-freezing method specified in GB/T 50082-2024 [34]. A fully automatic CLD-3 low-temperature freeze–thaw testing apparatus produced by Hebei Ourui Testing Instrument Co., Ltd. in Hebei Province, China, was utilized for this purpose, shown in Figure 5b. Each freeze–thaw cycle period consisted of 25 cycles. The mass loss rate, relative dynamic elastic modulus, and compressive strength loss rate served as evaluation indices. The physical and mechanical properties of each group of specimens were assessed after each freeze–thaw period. The arithmetic mean of test results from three specimens in each group was taken as the measured value for that group. The test for a group was terminated upon meeting any of the following criteria: mass loss rate exceeding 5%; relative dynamic elastic modulus falling below 60%; compressive strength loss rate exceeding 25%; or completion of 200 freeze–thaw cycles

#### 2.4.2. Microscopic Characterization

Specimens were sectioned into cubic samples with edge lengths ≤ 10 mm. These samples were ultrasonically cleaned in water to remove surface debris. After removal, the test surface was identified, sputter-coated with gold for approximately 30 s, and mounted on the sample stage for SEM-EDS analysis. Testing employed a ZEISS Sigma300 (Carl Zeiss AG, Oberkochen, Germany) field emission scanning electron microscope operating in secondary electron mode with an accelerating voltage of 10 kV and a working distance of 14.5 mm. SEM micrographs were acquired at magnifications of 50×, 1000×, and 10,000×. An Oxford Xplore30 energy dispersive X-ray spectrometer (Oxford Instruments, Oxford, UK) was used synchronously with the SEM observations. Point analysis was performed within a 100× magnification field of view. Spectra were collected for 60 s, enabling the EDS system to conduct qualitative and semi-quantitative elemental analysis of selected areas, yielding the distribution characteristics and weight percentages of major elements. The testing instruments are depicted in Figure 5c.

## 3. Results and Analysis

### 3.1. Experiment Results

The test results are presented in Table 4, which lists the average values and coefficients of variation (Cv) for slump, compressive strength, and splitting tensile strength of each RFCTSWC group. The coefficients of variation for slump test results are all below 3%, indicating good repeatability and concentrated data. The coefficients of variation for both compressive strength and splitting tensile strength are generally below 5%, with only Group 2’s compressive strength slightly above 5% at 5.62%. Overall, these values remain at an acceptable level, demonstrating good repeatability and reliability of the experimental results.

### 3.2. Orthogonal Test Analysis

**Figure 6:**
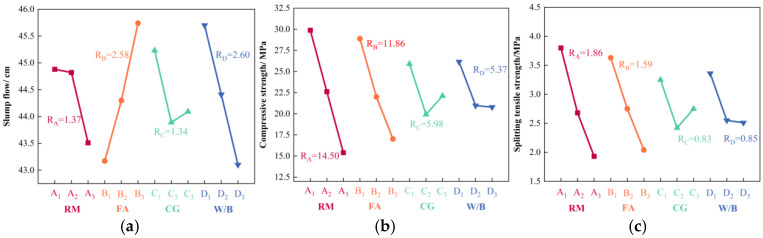
Influence laws of the four factors on each test index. (**a**) Slump. (**b**) Compressive strength. (**c**) Splitting tensile strength.

Statistical analysis was performed using SPSS 22.0 software to conduct range analysis and analysis of variance on the experimental results. This analysis determined the order of primary and secondary influence, as well as the optimal combination relationship, for the four factors—RM, FA, CGA, and W/B—on the test indicators. The analysis further examined the statistical impact of these four factors on each test indicator. The results of the range analysis and ANOVA are presented in Table 5 and Table 6, respectively, while Figure 6 illustrates the influence patterns of each factor on the slump, compressive strength, and splitting tensile strength.

#### 3.2.1. Slump Analysis

According to the range analysis results in Table 5, the order of influence of the four factors on slump was: W/B > FA > RM > CGA. The corresponding optimal level combination was D_1_B_3_A_1_C_1_, specifically a W/B of 0.45, FA content of 55%, RM content of 10%, and CGA content of 20%. The ANOVA results in Table 6 indicated that the effects of W/B and FA on slump were significant, while those of RM and CGA were not. This lack of significance for RM is attributed to the drastic decrease in slump (1.31 cm) observed when RM content reached 30%, which contributed 95.6% of the total range. The minimal differences between the first two RM levels diluted the overall effect in the ANOVA [35]. A similar dilution effect masking inter-group differences was observed for CGA. As shown in Figure 6a, slump exhibited an essentially linear increasing trend with FA content rising from 35% to 55%, indicating a positive influence of high FA content on slump. This is due to the micro-aggregate effect of FA; its spherical particles not only fill internal pores within the concrete but also reduce inter-particle friction, significantly enhancing fluidity [36]. Notably, slump did not increase with higher W/B. This counterintuitive result occurs because the superplasticizer ensures adequate fluidity at lower W/B, while excessively high ratios lead to surplus free water, reducing the cohesion of the paste and consequently impairing slump development [37].

#### 3.2.2. Compressive Strength Analysis

The compressive strength of RFCTSWC specimens varied significantly, ranging from 10 to 43 MPa. Range analysis in Table 5 revealed the order of influence of the four factors on compressive strength as: RM > FA > CGA > W/B. The optimal level combination was A_1_B_1_C_1_D_1_, corresponding to RM content of 10%, FA content of 35%, CGA content of 20%, and a W/B of 0.45, which yielded the maximum compressive strength. ANOVA results in Table 6 indicated that the effects of RM and FA on compressive strength were highly significant, while those of CGA and W/B were moderately significant. The *p*-value for RM was an order of magnitude lower than that for FA, signifying that RM content exerted the most pronounced influence on compressive strength, consistent with the range analysis results.

As illustrated in Figure 6b, compressive strength exhibited a downward trend with increasing RM content, FA content, and W/B, showing decreases of 14.50 MPa, 11.86 MPa, and 5.37 MPa, respectively. This indicates an inverse relationship between these factors and compressive strength development. The decline is attributable to RM’s low cementitious activity, where unreacted RM particles tend to agglomerate, disrupting the C-S-H gel structure [10]. Furthermore, increased RM and FA content directly reduces cement dosage, impeding the rate and quantity of C-S-H gel formation [38]. The reduced rate of strength decline at higher W/B (0.50–0.55) occurs because the concrete’s internal structure already exhibits high porosity within this range; further increases in W/B approach saturation in terms of densification impairment, resulting in a minimal strength reduction of merely 0.19 MPa. Conversely, compressive strength initially decreased and then increased with rising CGA content. This trend is likely due to optimized aggregate gradation at 60% CGA content compared to 20%, thus enhancing compactness between aggregates [39], leading to a mean compressive strength increase of 2.2 MPa.

#### 3.2.3. Splitting Tensile Strength Analysis

The splitting tensile strength of RFCTSWC specimens ranged from 1 to 6 MPa. Range analysis (Table 5) indicated the order of factor influence as: RM > FA > W/B > CGA, with the optimal combination A_1_B_1_D_1_C_1_ (RM: 10%, FA: 35%, W/B: 0.45, CGA: 20%) yielding maximum strength. ANOVA in Table 6 revealed highly significant effects of RM and FA content, and moderately significant effects of W/B and CGA on splitting tensile strength. This significance difference arises because increased RM and FA content reduce C-S-H gel formation, weakening matrix bonding, while splitting tensile strength critically depends on mortar matrix continuity [40]. As shown in Figure 6c, the influence trends of all factors on splitting tensile strength mirrored those for compressive strength: mean strength decreased with higher RM, FA, and W/B, while increasing CGA content caused an initial decrease followed by an increase.

### 3.3. Frost Resistance Durability

Given the highly significant influence of RM and FA dosages on the mechanical properties of concrete, three specimen groups with incrementally increasing RM and FA contents were selected for frost resistance testing: RF1 (10% RM, 35% FA), RF2 (20% RM, 45% FA), and RF3 (30% RM, 55% FA).

#### 3.3.1. Appearance Changes

**Figure 7:**
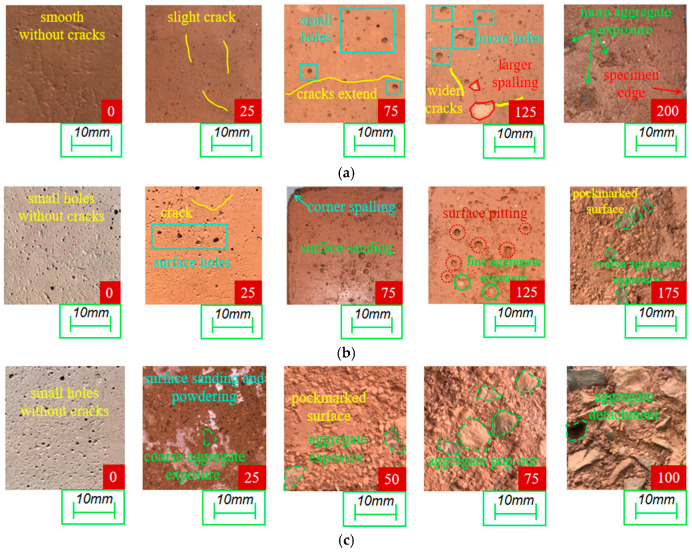
Appearance changes in the three groups of test blocks with the increase in freeze–thaw cycles. (**a**) Appearance change diagram of RF1 specimen. (**b**) Appearance change diagram of RF2 specimen. (**c**) Appearance change diagram of RF3 specimen.

Figure 7 illustrates the surface deterioration of the three specimen groups under varying freeze–thaw cycles. Figure 7a shows RF1 specimens developing surface microcracks after 25 cycles, with crack widths approaching 0.1 mm. After 75 cycles, capillary pores on the specimen surface enlarged due to dissolution, expanding from less than 1 mm to over 1 mm in diameter. By 125 cycles, localized flaking (area < 100 mm^2^) occurred, accompanied by widened surface cracks ranging between 0.5 and 1 mm. Upon reaching the specified 200 cycles, significant dusting and exposed coarse aggregates were observed, alongside enlarged areas of hydration product detachment. Crucially, the specimen edges showed no noticeable rounding, indicating RF1 specimens endured 200 cycles without excessive damage.

As depicted in Figure 7b, RF2 specimens primarily exhibited crack propagation and capillary pore development during initial freeze–thaw stages. After 75 cycles, slight dusting formed shallow pits exceeding 1 mm in diameter, accompanied by rounding of sharp edges. By 125 cycles, the surface became distinctly pockmarked with visible fine aggregates. At 175 cycles, extensive pockmark-like surface spalling and significant coarse aggregate exposure occurred. This exposure appeared one cycle period (25 freeze–thaw cycles) earlier than in RF1 and demonstrated markedly greater deterioration.

Surface changes in RF3 specimens (Figure 7c) revealed pronounced dusting and powdering after only 25 cycles, occurring 50 and 100 cycles earlier than in RF1 and RF2, respectively. Deterioration progressed significantly faster: severe pockmarks and distinct coarse aggregate exposure emerged by 50 cycles. Corresponding to Section 3.2.2 results, the mass loss rate reached 4.4% at this stage, approaching the 5% failure threshold. Specimens became structurally loose after 75 cycles, losing structural integrity with protruding coarse aggregates detaching from the mortar. By 100 cycles, coarse aggregates were dislodged entirely with extensive separation between aggregates and mortar.

In summary, damage progressed gradually in RF1 and RF2, though RF2 deteriorated faster and more severely. RF3 exhibited exponentially accelerated surface deterioration. This is attributed to: (1) Reduced cementitious phases from higher RM and FA content, weakening paste cohesion; (2) Increased Na^+^ and K^+^ dissolution from higher RM content, elevating internal alkalinity, which dissolves Ca^2+^ from C-S-H gel [41], damaging pore structure and accelerating failure. Thus, RM and FA content is inversely correlated with the onset of surface deterioration and positively correlated with its severity in the RFCTSWC—higher content leads to earlier initiation and more severe damage.

#### 3.3.2. Quality Loss Rate

**Figure 8:**
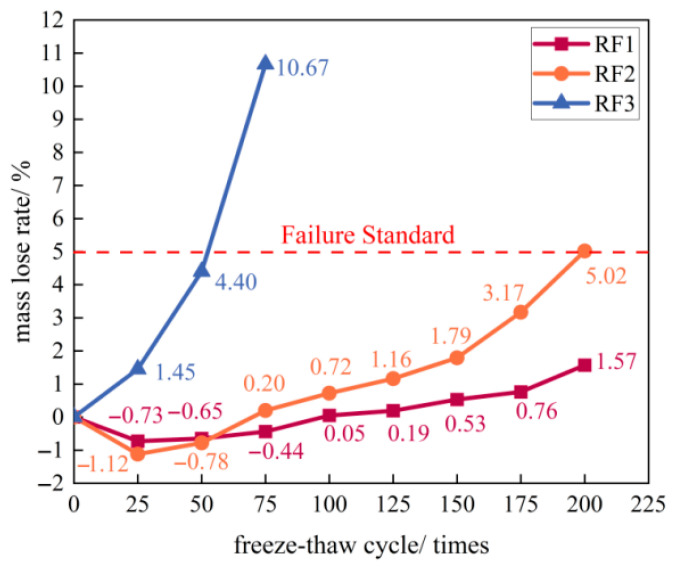
Variation in mass loss rate of the three specimen groups versus freeze–thaw cycles.

As shown in Figure 8, the mass loss ratio of the RF1 group exhibited an initial decrease followed by an increase, with the turning point occurring around 25 freeze–thaw cycles. The ratio remained negative until 75 cycles, attributable to water replacing air within the pores of the mortar and CGA, leading to mass gain [42]. During this phase, the development of micro-pores and surface cracks facilitated water absorption [43]. A mass loss ratio of 0.05% at 100 cycles corresponded with the onset of minor surface spalling. After 200 cycles, the mass loss ratio reached 1.57%, remaining below the failure threshold (5%).

The RF2 group also showed an initial decrease in mass loss ratio, reaching −1.12% at 25 cycles −0.39% lower than RF1 at the same stage. This greater mass gain stemmed from the higher CGA content (60% of coarse aggregate, triple that of RF1), resulting in higher porosity and water absorption. Subsequently, the mass loss ratio increased, reaching 0.2% by 75 cycles—a rate significantly faster than RF1, consistent with the earlier and more severe dusting observed in RF2. Rapid mass loss commenced after 150 cycles, coinciding with exposed coarse aggregates and debonding between aggregate and mortar. The mass loss ratio reached 5.02% at 200 cycles, exceeding the failure threshold.

The RF3 group displayed a continuous increase in mass loss ratio with freeze–thaw cycling, lacking the initial decrease phase seen in RF1 and RF2. The rate of mass loss accelerated exponentially every 25 cycles (5.80%, 11.80%, and 25.08%). The ratio reached 4.4% (approaching the 5% failure threshold) at 50 cycles and 10.67% (far exceeding the standard) at 75 cycles. This rapid deterioration corresponds to the early onset of significant dusting, powdering, and coarse aggregate exposure observed after just 25 cycles, progressing to severe spalling and mortar-aggregate detachment by 75 cycles.

#### 3.3.3. Loss Rate of Compressive Strength

**Figure 9:**
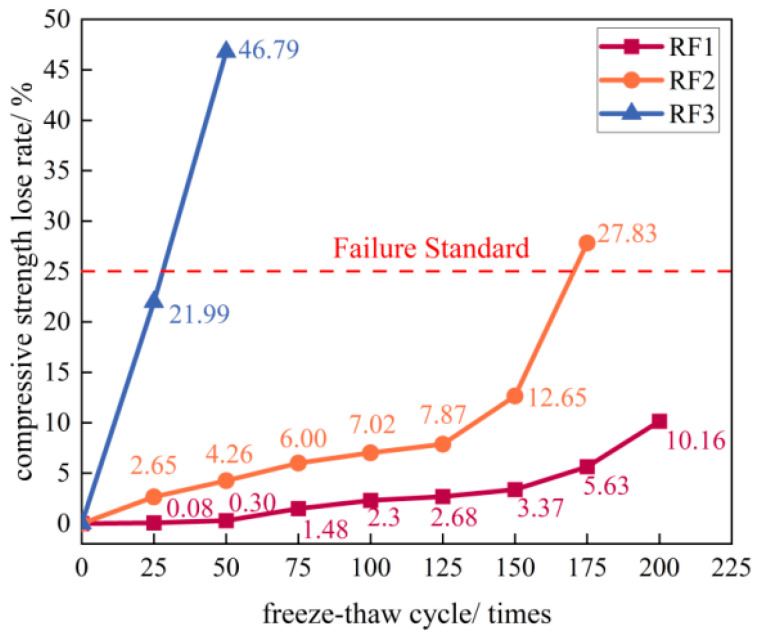
Variation in compressive strength loss rate of the three specimen groups versus freeze–thaw cycles.

As shown in Figure 9, the compressive strength loss rate of all three groups (RF1, RF2, RF3) exhibited increasing trends with rising freeze–thaw cycles. For RF1, the strength loss rate grew slowly (0.3%) during the first 50 cycles, increased by 1.18% at 75 cycles, and subsequently maintained a steady growth rate until 150 cycles, after which it accelerated, reaching 10.16% at 200 cycles—below the 25% failure threshold. Compared to RF1, RF2 consistently displayed a higher strength loss rate after each cycle, exhibiting a nonlinear increasing trend. Its loss rate grew steadily through 125 cycles but accelerated significantly thereafter, with increases of 4.78% and 15.18% at cycles 150 and 175, respectively, reaching the failure threshold at 175 cycles. RF3 exhibited a linear increase in strength loss rate, reaching 21.99% (approaching the 25% threshold) after only 25 cycles and 46.79% after 50 cycles—indicating nearly halved strength within two cycle periods. This further demonstrates that RM and FA incorporation promotes compressive strength loss development in the RFCTSWC, with higher dosages positively correlating with increased post-freeze–thaw strength deterioration.

#### 3.3.4. Relative Dynamic Elastic Modulus

**Figure 10:**
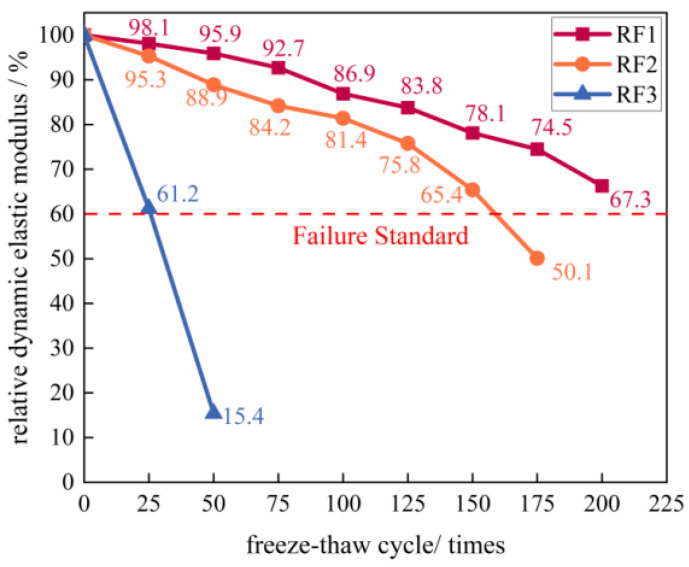
Variation in relative dynamic elastic modules of the three specimen groups versus freeze–thaw cycles.

Figure 10 illustrates the variation in relative dynamic elastic modulus (RDEM) of specimen groups RF1, RF2, and RF3 with increasing freeze–thaw cycles. The RDEM of all groups exhibited a declining trend. The descending rate at equivalent cycles followed the order RF3 > RF2 > RF1, indicating that higher contents of RM and CGA accelerated deterioration. For RF1, the RDEM decreased at a relatively constant rate throughout the testing period, reaching 67.3% after 200 cycles without attaining the failure threshold of 60%. The RDEM decline of RF2 was more pronounced during the initial stage and after 100 cycles, showing a nonlinear pattern over the entire process; it reached the failure threshold at 50.1% after 175 cycles. RF3 exhibited a near-linear decrease in RDEM, reaching 61.2% after only 25 cycles, close to the failure threshold. After 50 cycles, the RDEM plummeted to 15.4%, indicating the specimens had nearly failed. The primary reason for this phenomenon lies in the poorest micro-pore compactness of the RF3 specimens in Section 3.4.1. Since the relative dynamic elastic modulus is highly sensitive to internal pores, its deterioration intensified more rapidly under freeze–thaw loading [44]. This accounts for the more pronounced decline in relative dynamic elastic modulus compared to the strength loss rate at the same number of freeze–thaw cycles, and explains why the relative dynamic elastic modulus approached the failure criterion earlier than other properties across all three specimen groups during the freeze–thaw process.

#### 3.3.5. Quantitative Relationship Between Freeze–Thaw Damage and Physical and Mechanical Properties of RFCTSWC

**Figure 11:**
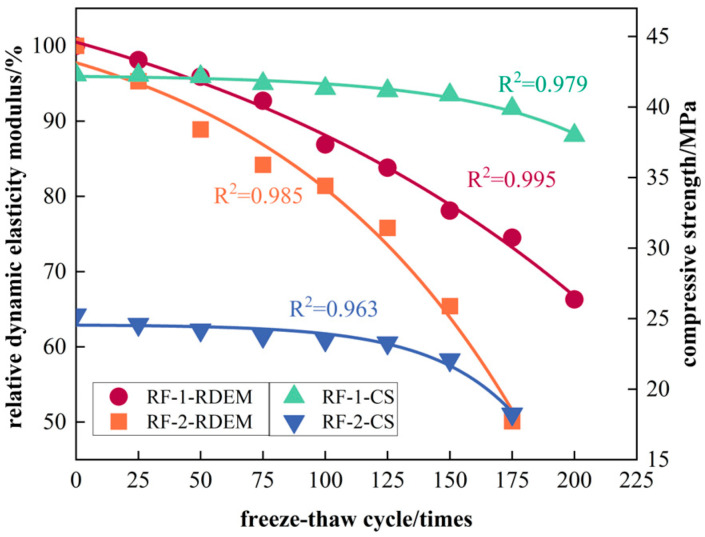
Fitting curve between freeze–thaw times and physical and mechanical properties of RFCTSWC.

Considering the heterogeneity and nonlinearity of concrete materials, an exponential decay model used in Equation (1) was adopted in this study to simulate the quantitative relationship between freeze–thaw damage and the physical-mechanical properties of RFCTSWC. The dependence of relative dynamic elastic modulus and compressive strength on freeze–thaw cycles is illustrated in Figure 11.

(1) $$y=A_{1}e^{-\frac{x}{t_{1}}}+y_{0}$$

where *A*_1_, *y*_0_, and *t*_1_ are fitting parameters.

Due to the limited availability of only two data points for the RF3 mix proportion, and to ensure the robustness of the modeling results, the aforementioned model was applied solely to the RF1 and RF2 mixes. The corresponding fitting results are presented in Table 7.

The results indicate that the decay in physical-mechanical properties of the RF2 group became significantly faster than that of RF1 starting from the mid-stage of freeze–thaw cycling (100 cycles). The goodness-of-fit (R^2^) for both mix proportions exceeded 0.95, demonstrating a strong nonlinear correlation between freeze–thaw damage and physical-mechanical properties in RFCTSWC, and validating the exponential decay model’s accuracy in characterizing the quantitative relationship between cycle number and performance degradation. Additionally, the initial RDEM and CS values of the RF2 specimens were lower than those of RF1, indicating that increased RM-FA content not only accelerates performance decay but also compromises the material’s initial properties. Although no decay model was established for RF3 due to data limitations, the analysis presented in Section 3.3.1, Section 3.3.2, Section 3.3.3 and Section 3.3.4 reveals that at high RM-FA content, freeze–thaw damage undergoes exponentially accelerated progression. This suggests the existence of a critical threshold level for RM-FA content regarding freeze–thaw damage susceptibility.

### 3.4. Microstructure Characterization

#### 3.4.1. Microscopic Morphology Characteristics

**Figure 12:**
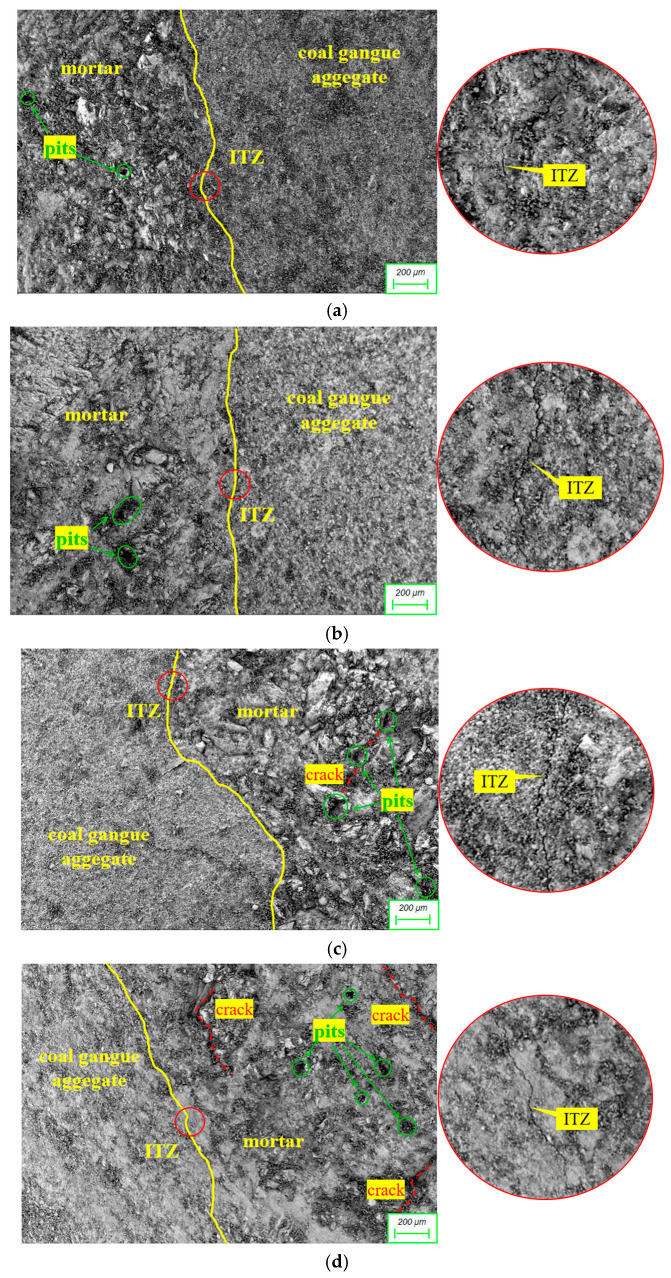

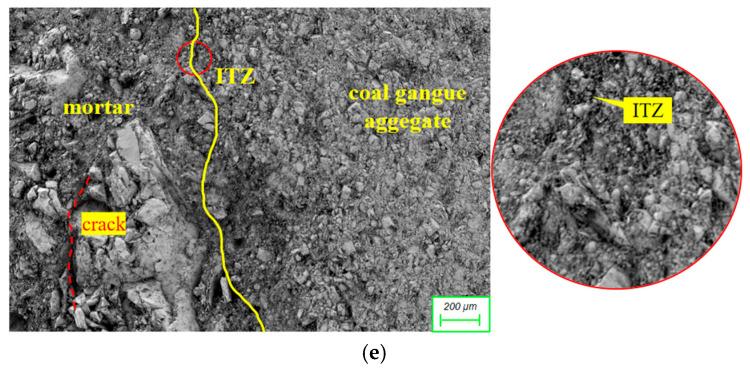
ITZ microscopic morphology of RFCTSWC under different combinations of red mud-fly ash dosage (50×). (**a**) specimen with 10% RM + 35% FA. (**b**) specimen with 10% RM + 45% FA. (**c**) specimen with 10% RM + 55% FA. (**d**) specimen with 20% RM + 45% FA. (**e**) specimen with 30% RM + 55% FA.

Figure 12 presents the micro-morphology at 50× magnification for RFCTSWC with different RM-FA incorporation ratios. Comparison of micrographs (a), (b), and (c) reveals that with a constant 10% RM content, increasing FA content led to a widening tendency of the interfacial transition zone (ITZ). Concurrently, the mortar matrix progressed from a dense structure with minor pits to larger, deeper pits accompanied by localized fragmentation, and finally to expanded fragmented areas interconnected by cracks. This indicates that elevated FA content detrimentally influences microstructural evolution when RM content is fixed, attributable to the delayed development of FA’s pozzolanic effect. Excessive FA incorporation results in unhydrated particles acting as internal defects, increasing ITZ width and enhancing pore connectivity [45]. Comparing micrographs (b) and (d) at a constant 45% FA content shows that increasing RM from 10% to 20% narrowed the ITZ. Although the mortar exhibited increased pitting and interconnected cracks, it maintained moderate overall integrity with limited porosity, suggesting RM effectively refines the RFCTSWC microstructure within a specific dosage range. Micrographs (c) and (e), both at 55% FA content but with RM contents of 10% and 30% respectively, demonstrate distinct microstructures: (c) retained a generally dense mortar structure with moderate fragmentation, while (e) displayed a highly porous, loose, and severely fragmented mortar. This highlights the amplified negative impact of increased RM content on mortar microstructure at high FA dosages. The high volumes of both FA and RM dilute the cementitious binder, reducing structural compactness and increasing porosity. This observation further validates the continuous decline in compressive and splitting tensile strength with increasing RM and FA content, as presented in Figure 5b,c.

**Figure 13:**
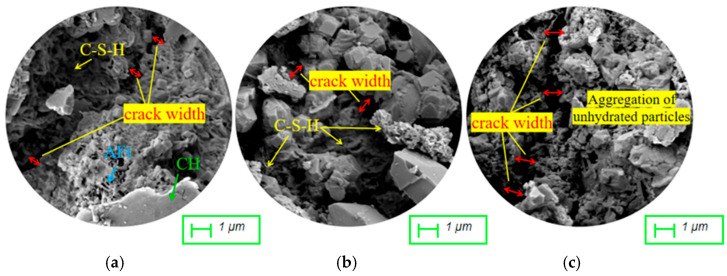

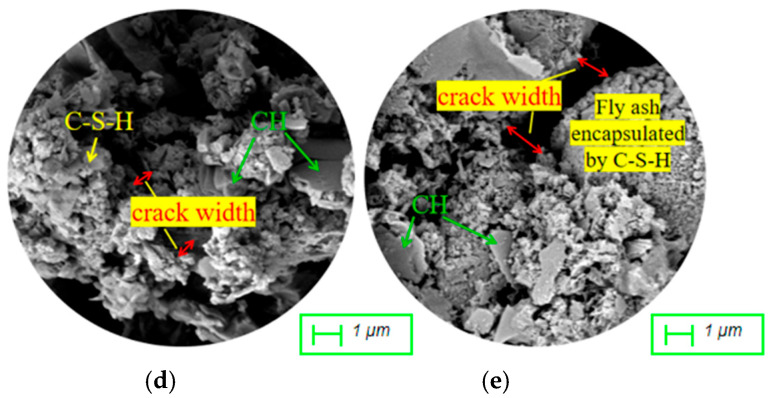
Microscopic morphology of mortar under the combination of red mud and fly ash dosage (10,000×). (**a**) 10% RM + 35% FA. (**b**) 10% RM + 45% FA. (**c**) 10% RM + 55% FA. (**d**) 20% RM + 45% FA. (**e**) 30% RM + 55% FA.

To further investigate the synergistic effects of RM and FA, the micro-morphology of mortar in RFCTSWC was examined at a higher magnification of 10.0 KX, as shown in Figure 13. Image (a) reveals a dense, uniformly distributed network of fibrous C-S-H gel with microcracks narrower than 0.5 μm. With increased FA content, image (b) shows reduced distribution of C-S-H gel, though still dense, accompanied by widened microcracks. Image (c) exhibits localized aggregation of unhydrated particles, resulting in a loose, porous structure with cracks widening to approximately 1 μm, confirming the detrimental impact of high FA dosage on microstructural densification at constant RM content. Comparing images (b) and (d) reveals an increased quantity of C-S-H gel in the microstructure without significant crack propagation, while image (d) demonstrates superior C-S-H gel distribution and narrower cracks relative to image (c). Correlating with macro-mechanical tests, the compressive strengths of groups (b), (c), and (d) were 24.87 MPa, 21.90 MPa, and 24.96 MPa, respectively. This indicates an optimal blending ratio for RM and FA, where the combination of 20% RM and 45% FA yields superior macro-micro performance compared to 10% RM + 45% FA or 10% RM + 55% FA. Image (e), with RM content increased to 30%, shows FA particles enveloped by gel but exhibits larger interstructural cracks and higher porosity than image (c).

#### 3.4.2. SEM-EDS Element Distribution

**Figure 14:**
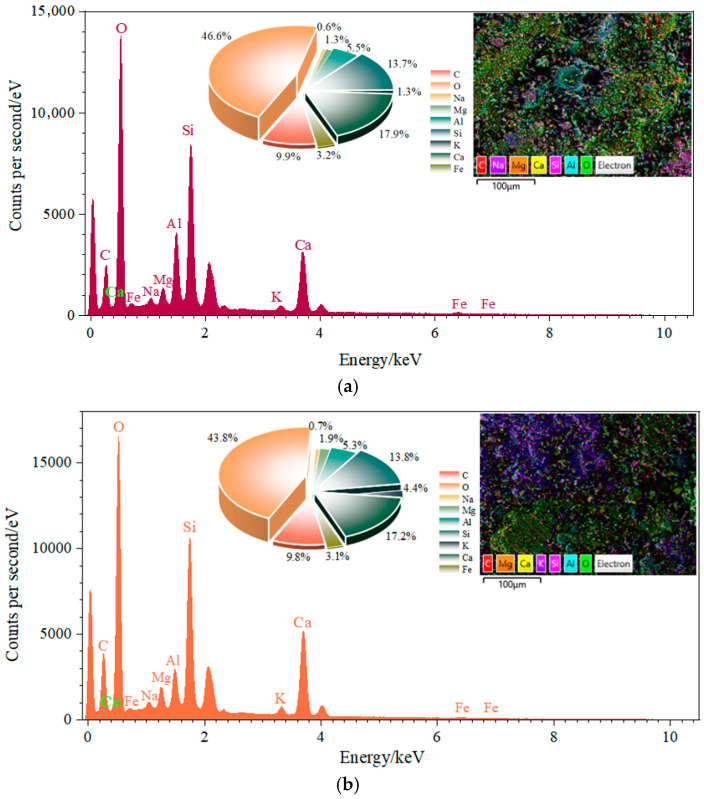

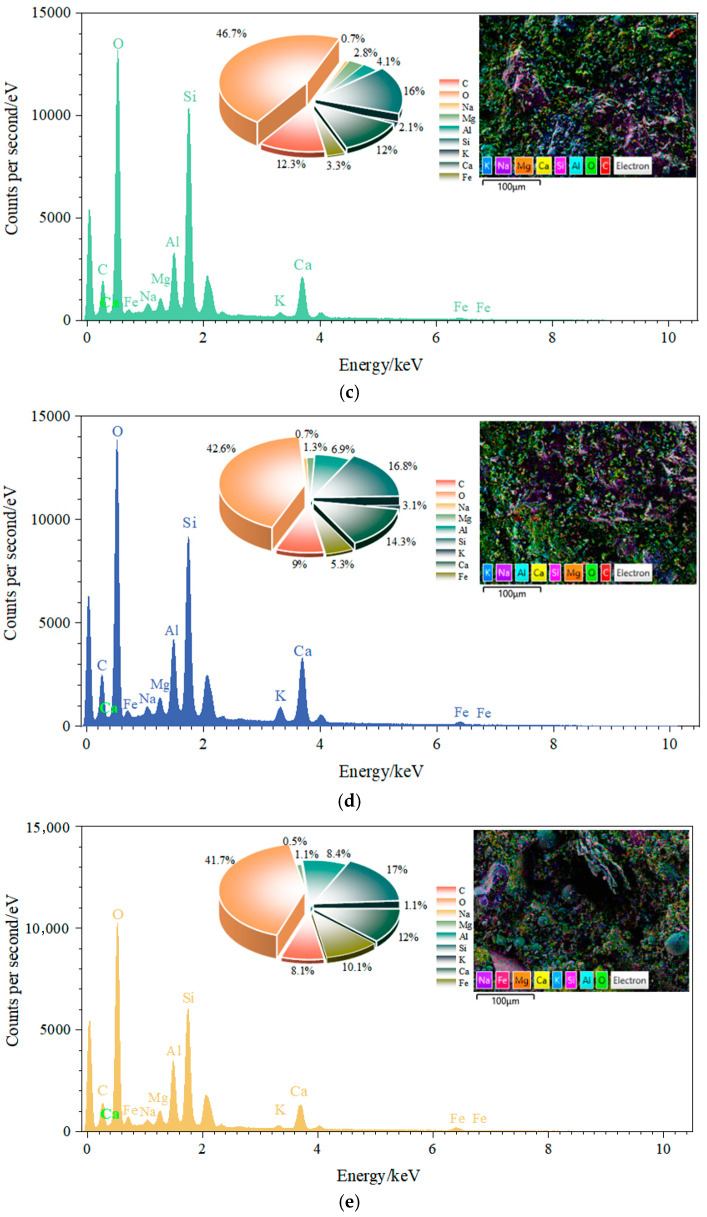
EDS energy spectrum analysis of RFCTSWC under different combinations of red mud and fly ash dosages. (**a**) EDS spectrum of specimen with 10% RM + 35% FA. (**b**) EDS spectrum of specimen with 10% RM + 45% FA. (**c**) EDS spectrum of specimen with 10% RM + 55% FA. (**d**) EDS spectrum of specimen with 20% RM + 45% FA. (**e**) EDS spectrum of specimen with 30% RM + 55% FA.

As shown in Figure 14, the Ca/Si ratios decreased from 1.309 to 1.243 and 0.747 as FA content increased from 35% to 55% at a constant RM content of 10%. This declining trend results from FA’s high SiO_2_ content, which elevates silicon concentration in the system. Concurrently, higher FA proportions reduce the cement fraction within the binder, diminishing the Ca supply derived from cement’s CaO. Consequently, the Ca/Si ratio decreases more substantially with greater FA incorporation [46].

At 45% FA content, the Ca/Si ratios measured 1.243 and 0.853 with RM contents of 10% and 20%, respectively, while the Al/Si ratios were 0.387 and 0.413. Corresponding to 55% FA content, Ca/Si ratios reached 0.747 and 0.707 with 10% and 30% RM, while Al/Si ratios were 0.259 and 0.493. These data indicate that increasing RM content at fixed FA levels reduces the Ca/Si ratio but elevates the Al/Si ratio. This behavior arises because higher RM proportions decrease the cement fraction (the primary Ca source) while introducing additional Al_2_O_3_ from RM.

## 4. Discussion

### 4.1. Comparative Analysis with Existing Studies

Current research has incorporated various solid wastes into concrete materials, including red mud, coal gangue, blast furnace slag, desulfurization gypsum, fly ash, and iron tailings, either individually or in combination. Studies indicate that when solid waste content exceeds 50–70%, structural performance negatively correlates with waste content [47], and strength fails to reach benchmark concrete levels [48]. The RM-FA-CGA ternary concrete system developed in this study demonstrates that concrete performance requirements can still be met at a total solid waste incorporation rate of 85%. By quantifying the exponential decay model between solid waste content and frost damage rate and analyzing the micro-mechanisms of multi-waste synergy, this research confirms the existence of a synergistic threshold for solid waste content. At this threshold point, a balance between macroscopic strength and microscopic structure is achieved, providing a new theoretical basis for designing multi-solid-waste systems.

### 4.2. Interrelationships Between Key Properties

Balancing property trade-offs presents a core challenge in solid waste concrete design. This study reveals an antagonistic relationship between workability and mechanical properties: while high FA content enhances RFCTSWC fluidity through the microsphere effect, excessive addition causes strength regression. This mechanism aligns with observations in silica fume-modified concrete where strength gain accompanies fluidity loss [49]. Additionally, a synergistic decay relationship exists between mechanical properties and frost resistance durability. Increased RM and FA content promotes agglomeration of unhydrated particles, increasing pore connectivity and destabilizing C-S-H gel structure, ultimately reducing strength and accelerating freeze–thaw damage.

### 4.3. Engineering Applications and Sustainability Implications

Performance interrelationships and threshold effects indicate that the threshold mixture withstood 175–200 freeze–thaw cycles, meeting structural requirements for most cold-region applications [50], making it suitable for bridges and high-rise buildings. The 85% solid waste content also delivers significant economic and environmental benefits. However, exceeding the synergistic threshold reduces strength and durability, shortening service life while increasing maintenance or demolition costs. Consequently, lifecycle carbon emissions may surpass those of ordinary concrete. Future research should focus on solid waste activation and nano-modification technologies to overcome barriers between waste content and performance enhancement.

## 5. Conclusions

This study employs an orthogonal experimental design to investigate the effects of RM content, FA content, CGA proportion, and W/B on the properties of RFCTSWC, combining freeze–thaw cycling tests with microstructural characterization to elucidate its performance evolution mechanisms. The following conclusions were drawn through analysis of macro-micro results:

(1) Slump is primarily governed by W/B, while FA’s micro-aggregate effect notably enhances fluidity. Compressive and split tensile strengths are most significantly affected by RM content. Increasing the combined dosage of RM and FA reduces cementitious hydration products, leading to a linear decline in mechanical properties. Optimal mechanical performance is achieved with low RM and FA dosages synergistically combined with 20% CGA and a W/B of 0.45.
(2) Increasing the combined RM and FA dosage reduces frost resistance durability. Specimens in group RF3 (30% RM + 55% FA) exhibited significant surface deterioration 100 and 50 freeze–thaw cycles earlier than groups RF1 (10% RM + 35% FA) and RF2 (20% RM + 45% FA), respectively, with accelerated deterioration rates. After 200 cycles, RF1 specimens showed no durability indicators reaching failure thresholds. RF2 specimens reached failure thresholds between cycles 175 and 200, while RF3 specimens approached thresholds in mass loss, strength loss, and relative dynamic modulus of elasticity as early as cycle 25th. An established decay model linking freeze–thaw damage to physical-mechanical properties indicates that higher RM-FA dosages not only accelerate performance degradation but also weaken the material’s initial properties.
(3) A significant synergistic effect exists between the combined RM-FA dosage and the micro-morphology/mechanical properties of the concrete. At fixed RM content, increasing FA content reduces C-S-H gel formation, enhances pore connectivity, and lowers the Ca/Si ratio. At fixed FA content, increasing RM content exacerbates structural fragmentation and elevates the Al/Si ratio. A synergistic threshold occurs at 20% RM and 45% FA, characterized by uniformly distributed C-S-H gel and narrow cracks, corresponding to a superior compressive strength of 24.96 MPa compared to other blends (10% RM + 45% FA; 10% RM + 55% FA). However, excessive incorporation (30% RM + 55% FA) causes localized aggregation of unhydrated particles and increased porosity, validating the continuous decline in macroscopic strength.
(4) Synergistic complementarity among solid wastes was observed, but excellent concrete performance requires low-to-medium solid waste dosages. Future research should explore targeted strategies like raw material activation, nano-modification, or fiber reinforcement to balance the conflict between solid waste content and performance, thereby promoting the high-value utilization of industrial solid wastes in low-carbon building materials.

## Figures and Tables

**Table 1:** Chemical compositions of raw materials.

Raw Materials	Chemical Composition (%)								
	Al_2_O_3_	SiO_2_	CaO	Fe_2_O_3_	Na_2_O	K_2_O	MgO	TiO_2_	LOI
Red mud	16.45	12.36	2.56	50.35	8.10	0.22	-	1.78	8.18
Fly ash	28.57	48.15	6.68	6.36	2.57	1.21	0.83	0.85	4.78
Cement	6.68	18.24	65.15	2.88	0.68	0.57	4.25	-	1.55

**Table 2:** Sand specifications.

Category	Fineness Modulus	Close Packing Density (kg/m^3^)	Packing Density (kg/m^3^)	Relative Density (kg/m^3^)	Rock Content (%)	Particle Gradation
medium sand	2.6	1847.5	1635	2600	11.95	Zone Ⅱ

**Table 3:** Mix proportions design table.

Sample	Factor Level Value				Proportion of RFCTSWC (kg/m^3^)							
	RM	FA	CGA	W/B	RM	Cement	CGA	WRA	Sand	Gravel Aggregate	FA	Water
1	10%	35%	20%	0.45	39.68	213.50	227.38	4.76	667.70	909.51	138.89	178.58
2	10%	45%	40%	0.5	35.72	155.96	697.14	4.76	682.38	464.76	160.72	178.58
3	10%	55%	60%	0.55	35.72	105.63	472.94	4.76	694.39	709.41	178.58	178.58
4	20%	35%	40%	0.55	64.94	141.35	709.41	4.76	694.39	427.94	113.64	178.58
5	20%	45%	60%	0.45	79.37	134.13	454.76	4.76	667.70	682.14	178.58	178.58
6	20%	55%	20%	0.5	71.43	84.53	232.38	4.76	682.38	929.51	196.43	178.58
7	30%	35%	60%	0.5	107.15	120.24	464.76	4.76	682.38	697.14	125.00	178.58
8	30%	45%	20%	0.55	97.40	76.41	236.47	4.76	694.39	945.88	146.11	178.58
9	30%	55%	40%	0.45	119.05	54.77	682.14	4.76	667.70	454.76	218.26	178.58

Notes: WRA stands for Water Reducing Agent, the dosage is 0.2%.

**Table 4:** Experiment Results.

Sample	Slump		Compressive Strength		Splitting Tensile Strength	
	Average Value (cm)	Cv (%)	Average Value (cm)	Cv (%)	Average Value (cm)	Cv (%)
1	19.8	1.05	42.91	1.70	5.62	2.98
2	18.3	1.38	24.87	5.62	3.10	4.39
3	18.6	2.69	21.90	1.67	2.67	3.93
4	15.8	2.86	24.28	3.58	2.82	4.60
5	19.7	1.34	24.96	1.11	3.13	3.68
6	21.0	0.48	18.59	3.10	2.11	1.36
7	16.0	2.20	19.46	1.94	2.44	1.41
8	16.9	1.70	16.15	3.01	2.03	4.58
9	19.6	1.28	10.57	0.97	1.33	4.10

**Table 5:** Range analysis of workability and mechanical properties of RFCTSWC.

Test Index	Range Analysis	Factor				Optimum Level
		k_1_	k_2_	k_3_	R	
Slump	A	18.88	18.82	17.51	1.37	1
	B	17.17	18.30	19.74	2.58	3
	C	19.23	17.89	18.09	1.34	1
	D	19.70	18.41	17.10	2.60	1
	Influence degree: D > B > A >C					
Compressive strength	A	29.89	22.61	15.39	14.50	1
	B	28.88	21.99	17.02	11.86	1
	C	25.89	19.91	22.11	5.98	1
	D	26.15	20.97	20.78	5.37	1
	Influence degree: A > B > C > D					
Splitting tensile strength	A	3.80	2.69	1.93	1.86	1
	B	3.63	2.75	2.04	1.59	1
	C	3.25	2.42	2.75	0.83	1
	D	3.36	2.55	2.51	0.85	1
	Influence degree: A > B > D > C					

**Table 6:** Variance analysis of workability and mechanical properties of RFCTSWC.

Test Index	Source of Variance	Sum of Squares of Deviation	Degree of Freedom	Mean Square	F Value	*p* Value	Significant
Slump	A	10.7696	2	5.3848	2.9587	0.0775	-
	B	30.0474	2	15.0237	8.2548	0.0029	*
	C	9.4719	2	4.7359	2.6022	0.1017	-
	D	30.4207	2	15.2104	8.3573	0.0027	*
Compressive strength	A	946.0597	2	473.0299	56.6330	1.7143 × 10^−8^	**
	B	638.1683	2	319.0842	38.2020	3.3307 × 10^−7^	**
	C	164.6754	2	82.3377	9.8578	0.0013	*
	D	166.9952	2	83.4976	9.9967	0.0012	*
Splitting tensile strength	A	15.8440	2	7.9220	21.7790	1.5627 × 10^−5^	**
	B	11.4747	2	5.7373	15.7730	0.0001	**
	C	3.1591	2	1.5796	4.3425	0.0289	*
	D	4.1683	2	2.0842	5.7298	0.0119	*

Notes: * means significant; ** means highly significant; - means non-significant.

**Table 7:** Fitting results of freeze–thaw damage and physical and mechanical properties under three dosage combinations.

Dosage Combination	Fitting Relation	Fitting Result			
		*A*_1_	*y*_0_	*t*_1_	R^2^
RF1	RDEM-FTc	−16.98327	117.48665	−182.64386	0.995
	CS-FTc	−0.09108	42.25935	−52.3791	0.979
RF2	RDEM-FTc	−9.83674	107.60959	−100.57775	0.985
	CS-FTc	−0.03134	24.56594	−33.10658	0.963

Note: RDEM stands for relative dynamic elastic modules; CS stands for compressive strength; FTc stands for freeze–thaw cycle times.

## Data Availability

The original contributions presented in this study are included in the article. Further inquiries can be directed to the corresponding author.
